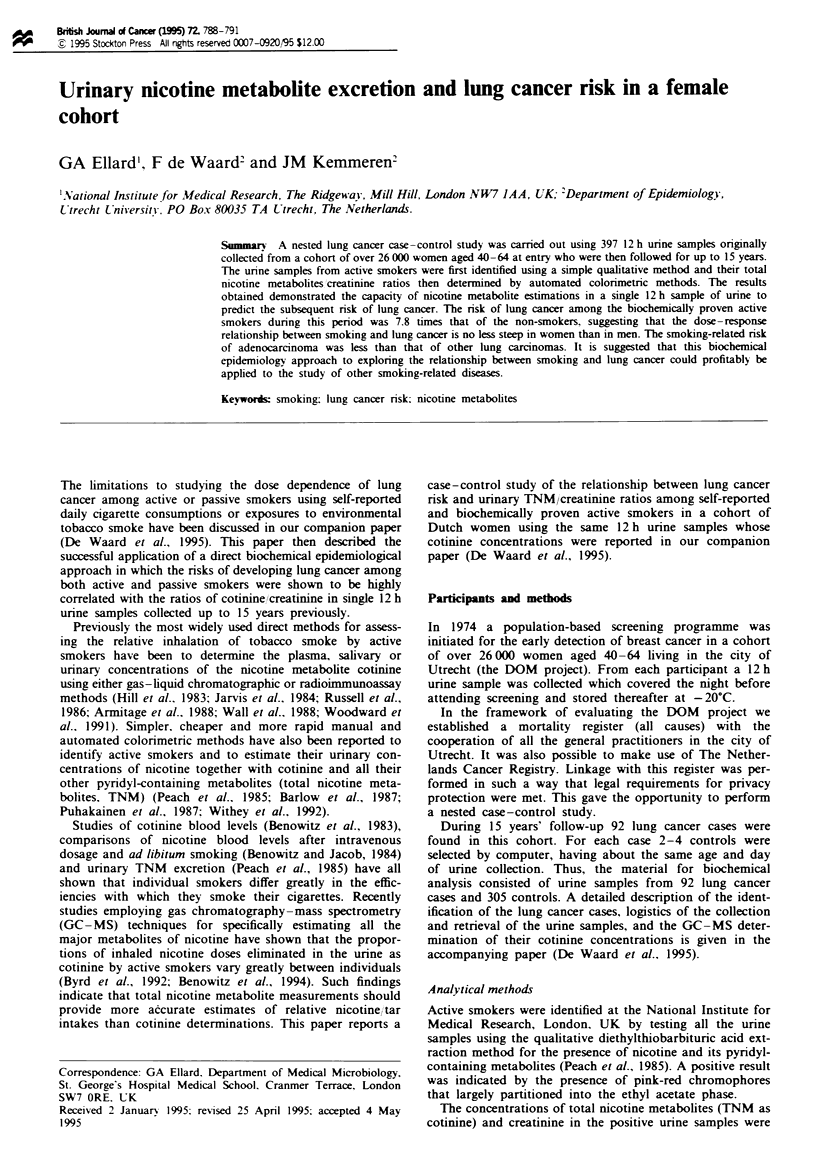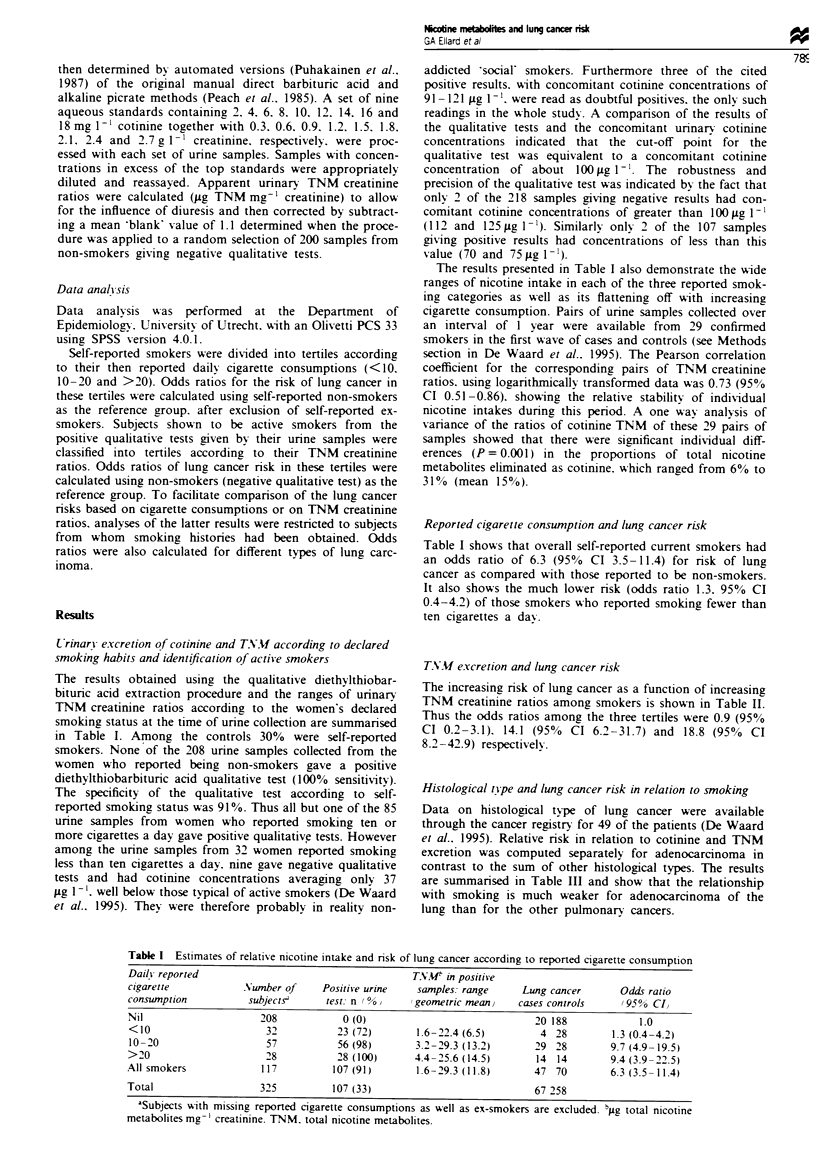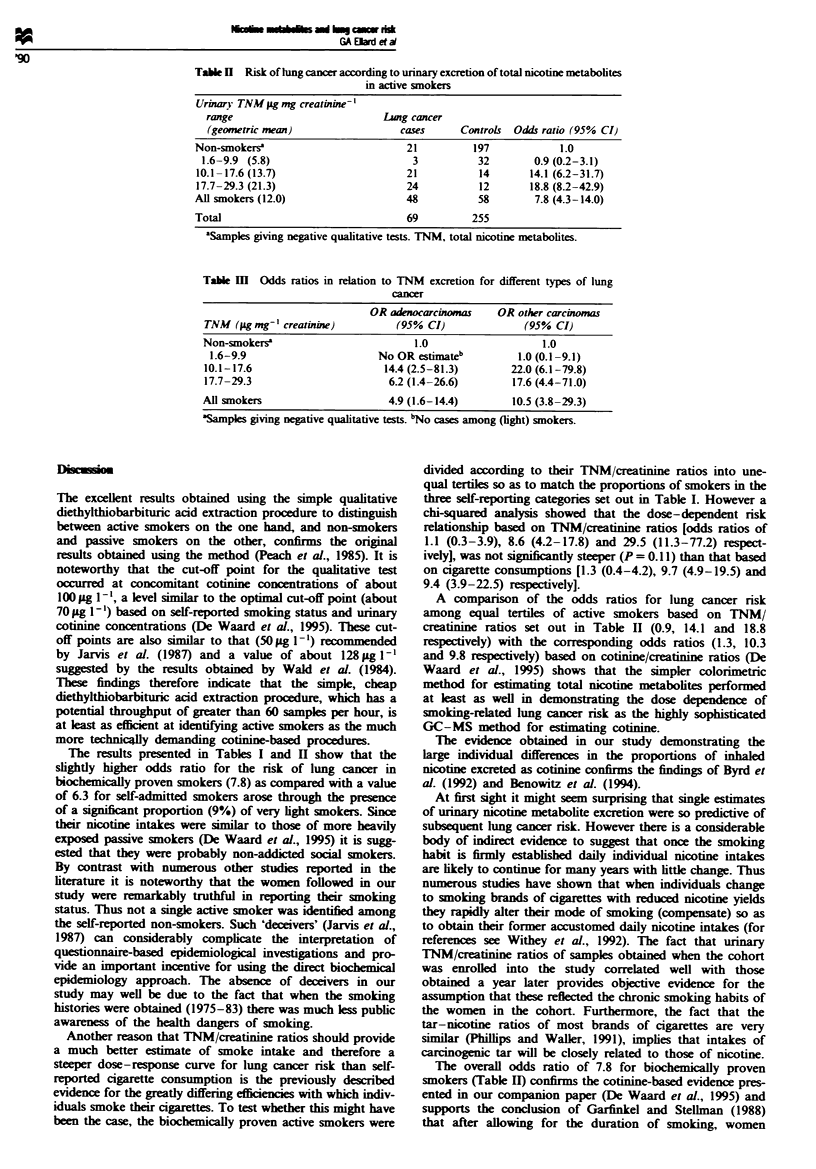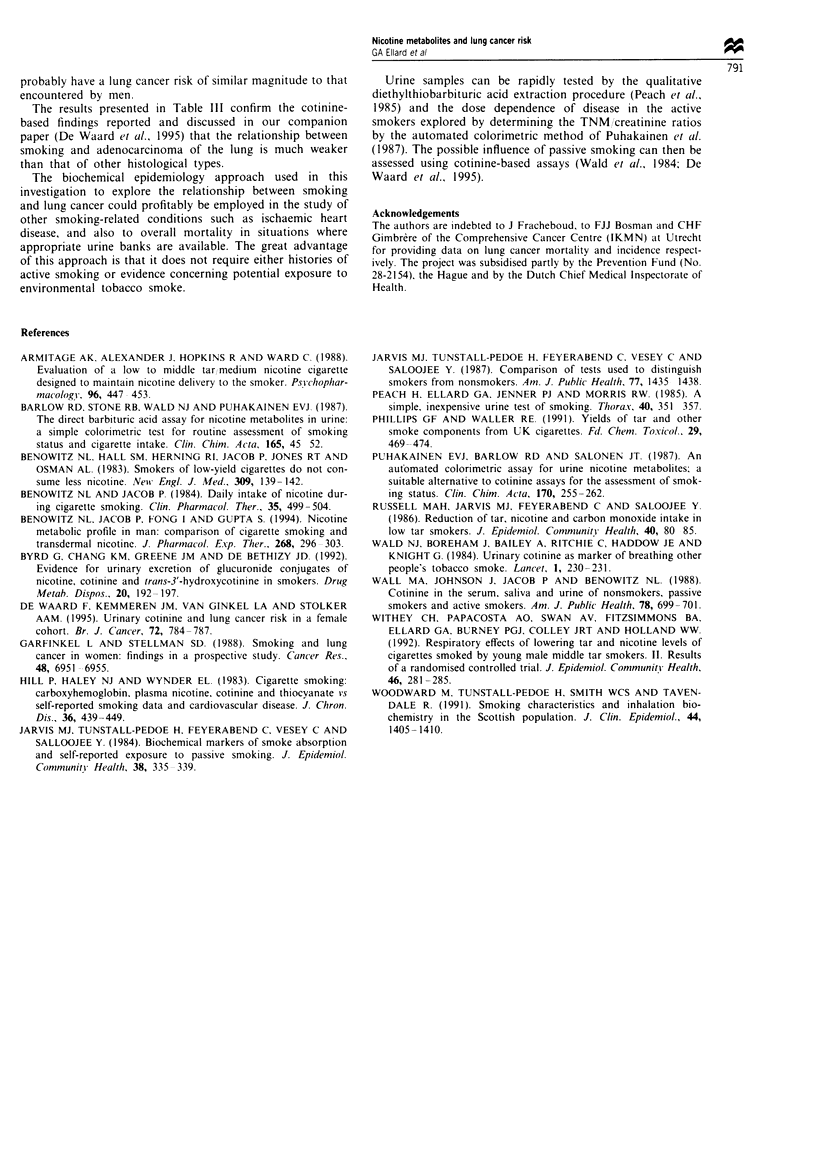# Urinary nicotine metabolite excretion and lung cancer risk in a female cohort.

**DOI:** 10.1038/bjc.1995.412

**Published:** 1995-09

**Authors:** G. A. Ellard, F. de Waard, J. M. Kemmeren

**Affiliations:** National Institute for Medical Research, Mill Hill, London, UK.

## Abstract

A nested lung cancer case-control study was carried out using 397 12 h urine samples originally collected from a cohort of over 26,000 women aged 40-64 at entry who were then followed for up to 15 years. The urine samples from active smokers were first identified using a simple qualitative method and their total nicotine metabolites/creatinine ratios then determined by automated colorimetric methods. The results obtained demonstrated the capacity of nicotine metabolite estimations in a single 12 h sample of urine to predict the subsequent risk of lung cancer. The risk of lung cancer among the biochemically proven active smokers during this period was 7.8 times that of the non-smokers, suggesting that the dose-response relationship between smoking and lung cancer is no less step in women than in men. The smoking-related risk of adenocarcinoma was less than that of other lung carcinomas. It is suggested that this biochemical epidemiology approach to exploring the relationship between smoking and lung cancer could profitably be applied to the study of other smoking-related diseases.


British Journal of Cancer (1995) 72, 788–791

© 1995 Stockton Press All rights reserved 0007–0920/95 $12.00

### Urinary nicotine metabolite excretion and lung cancer risk in a female cohort

GA Ellard[1], F de Waard[2] and JM Kemmeren[2]

[1]*National Institute for Medical Research, The Ridgeway, Mill Hill, London NW7 1AA, UK;* [2]*Department of Epidemiology, Utrecht University, PO Box 80035 TA Utrecht, The Netherlands.*

**Summary** A nested lung cancer case–control study was carried out using 397 12 h urine samples originally collected from a cohort of over 26 000 women aged 40–64 at entry who were then followed for up to 15 years. The urine samples from active smokers were first identified using a simple qualitative method and their total nicotine metabolites/creatinine ratios then determined by automated colorimetric methods. The results obtained demonstrated the capacity of nicotine metabolite estimations in a single 12 h sample of urine to predict the subsequent risk of lung cancer. The risk of lung cancer among the biochemically proven active smokers during this period was 7.8 times that of the non-smokers, suggesting that the dose–response relationship between smoking and lung cancer is no less steep in women than in men. The smoking-related risk of adenocarcinoma was less than that of other lung carcinomas. It is suggested that this biochemical epidemiology approach to exploring the relationship between smoking and lung cancer could profitably be applied to the study of other smoking-related diseases.

**Keywords:** smoking; lung cancer risk; nicotine metabolites

The limitations to studying the dose dependence of lung cancer among active or passive smokers using self-reported daily cigarette consumptions or exposures to environmental tobacco smoke have been discussed in our companion paper (De Waard *et al.*, 1995). This paper then described the successful application of a direct biochemical epidemiological approach in which the risks of developing lung cancer among both active and passive smokers were shown to be highly correlated with the ratios of cotinine/creatinine in single 12 h urine samples collected up to 15 years previously.

Previously the most widely used direct methods for assessing the relative inhalation of tobacco smoke by active smokers have been to determine the plasma, salivary or urinary concentrations of the nicotine metabolite cotinine using either gas–liquid chromatographic or radioimmunoassay methods (Hill *et al.*, 1983; Jarvis *et al.*, 1984; Russell *et al.*, 1986; Armitage *et al.*, 1988; Wall *et al.*, 1988; Woodward *et al.*, 1991). Simpler, cheaper and more rapid manual and automated colorimetric methods have also been reported to identify active smokers and to estimate their urinary concentrations of nicotine together with cotinine and all their other pyridyl-containing metabolites (total nicotine metabolites, TNM) (Peach *et al.*, 1985; Barlow *et al.*, 1987; Puhakainen *et al.*, 1987; Withey *et al.*, 1992).

Studies of cotinine blood levels (Benowitz *et al.*, 1983), comparisons of nicotine blood levels after intravenous dosage and *ad libitum* smoking (Benowitz and Jacob, 1984) and urinary TNM excretion (Peach *et al.*, 1985) have all shown that individual smokers differ greatly in the efficiencies with which they smoke their cigarettes. Recently studies employing gas chromatography–mass spectrometry (GC–MS) techniques for specifically estimating all the major metabolites of nicotine have shown that the proportions of inhaled nicotine doses eliminated in the urine as cotinine by active smokers vary greatly between individuals (Byrd *et al.*, 1992; Benowitz *et al.*, 1994). Such findings indicate that total nicotine metabolite measurements should provide more accurate estimates of relative nicotine/tar intakes than cotinine determinations. This paper reports a case–control study of the relationship between lung cancer risk and urinary TNM/creatinine ratios among self-reported and biochemically proven active smokers in a cohort of Dutch women using the same 12 h urine samples whose cotinine concentrations were reported in our companion paper (De Waard *et al.*, 1995).

### Participants and methods

In 1974 a population-based screening programme was initiated for the early detection of breast cancer in a cohort of over 26 000 women aged 40–64 living in the city of Utrecht (the DOM project). From each participant a 12 h urine sample was collected which covered the night before attending screening and stored thereafter at $-20^{\circ}C$.

In the framework of evaluating the DOM project we established a mortality register (all causes) with the cooperation of all the general practitioners in the city of Utrecht. It was also possible to make use of The Netherlands Cancer Registry. Linkage with this register was performed in such a way that legal requirements for privacy protection were met. This gave the opportunity to perform a nested case–control study.

During 15 years' follow-up 92 lung cancer cases were found in this cohort. For each case 2–4 controls were selected by computer, having about the same age and day of urine collection. Thus, the material for biochemical analysis consisted of urine samples from 92 lung cancer cases and 305 controls. A detailed description of the identification of the lung cancer cases, logistics of the collection and retrieval of the urine samples, and the GC–MS determination of their cotinine concentrations is given in the accompanying paper (De Waard *et al.*, 1995).

### *Analytical methods*

Active smokers were identified at the National Institute for Medical Research, London, UK by testing all the urine samples using the qualitative diethylthiobarbituric acid extraction method for the presence of nicotine and its pyridyl-containing metabolites (Peach *et al.*, 1985). A positive result was indicated by the presence of pink-red chromophores that largely partitioned into the ethyl acetate phase.

The concentrations of total nicotine metabolites (TNM as cotinine) and creatinine in the positive urine samples were

Correspondence: GA Ellard, Department of Medical Microbiology, St. George's Hospital Medical School, Cranmer Terrace, London SW7 0RE, UK
Received 2 January 1995; revised 25 April 1995; accepted 4 May 1995

then determined by automated versions (Puhakainen et al., 1987) of the original manual direct barbituric acid and alkaline picrate methods (Peach et al., 1985). A set of nine aqueous standards containing 2, 4, 6, 8, 10, 12, 14, 16 and 18 mg $l^{-1}$ cotinine together with 0.3, 0.6, 0.9, 1.2, 1.5, 1.8, 2.1, 2.4 and 2.7 g $l^{-1}$ creatinine, respectively, were processed with each set of urine samples. Samples with concentrations in excess of the top standards were appropriately diluted and reassayed. Apparent urinary TNM/creatinine ratios were calculated (μg TNM $mg^{-1}$ creatinine) to allow for the influence of diuresis and then corrected by subtracting a mean 'blank' value of 1.1 determined when the procedure was applied to a random selection of 200 samples from non-smokers giving negative qualitative tests.

### *Data analysis*

Data analysis was performed at the Department of Epidemiology, University of Utrecht, with an Olivetti PCS 33 using SPSS version 4.0.1.

Self-reported smokers were divided into tertiles according to their then reported daily cigarette consumptions (<10, 10-20 and >20). Odds ratios for the risk of lung cancer in these tertiles were calculated using self-reported non-smokers as the reference group, after exclusion of self-reported ex-smokers. Subjects shown to be active smokers from the positive qualitative tests given by their urine samples were classified into tertiles according to their TNM/creatinine ratios. Odds ratios of lung cancer risk in these tertiles were calculated using non-smokers (negative qualitative test) as the reference group. To facilitate comparison of the lung cancer risks based on cigarette consumptions or on TNM/creatinine ratios, analyses of the latter results were restricted to subjects from whom smoking histories had been obtained. Odds ratios were also calculated for different types of lung carcinoma.

### Results

### *Urinary excretion of cotinine and TNM according to declared smoking habits and identification of active smokers*

The results obtained using the qualitative diethylthiobarbituric acid extraction procedure and the ranges of urinary TNM/creatinine ratios according to the women's declared smoking status at the time of urine collection are summarised in Table I. Among the controls 30% were self-reported smokers. None of the 208 urine samples collected from the women who reported being non-smokers gave a positive diethylthiobarbituric acid qualitative test (100% sensitivity). The specificity of the qualitative test according to self-reported smoking status was 91%. Thus all but one of the 85 urine samples from women who reported smoking ten or more cigarettes a day gave positive qualitative tests. However among the urine samples from 32 women reported smoking less than ten cigarettes a day, nine gave negative qualitative tests and had cotinine concentrations averaging only 37 μg $l^{-1}$, well below those typical of active smokers (De Waard et al., 1995). They were therefore probably in reality non-addicted 'social' smokers. Furthermore three of the cited positive results, with concomitant cotinine concentrations of 91-121 μg $l^{-1}$, were read as doubtful positives, the only such readings in the whole study. A comparison of the results of the qualitative tests and the concomitant urinary cotinine concentrations indicated that the cut-off point for the qualitative test was equivalent to a concomitant cotinine concentration of about 100 μg $l^{-1}$. The robustness and precision of the qualitative test was indicated by the fact that only 2 of the 218 samples giving negative results had concomitant cotinine concentrations of greater than 100 μg $l^{-1}$ (112 and 125 μg $l^{-1}$). Similarly only 2 of the 107 samples giving positive results had concentrations of less than this value (70 and 75 μg $l^{-1}$).

The results presented in Table I also demonstrate the wide ranges of nicotine intake in each of the three reported smoking categories as well as its flattening off with increasing cigarette consumption. Pairs of urine samples collected over an interval of 1 year were available from 29 confirmed smokers in the first wave of cases and controls (see Methods section in De Waard et al., 1995). The Pearson correlation coefficient for the corresponding pairs of TNM/creatinine ratios, using logarithmically transformed data was 0.73 (95% CI 0.51-0.86), showing the relative stability of individual nicotine intakes during this period. A one way analysis of variance of the ratios of cotinine/TNM of these 29 pairs of samples showed that there were significant individual differences ($P = 0.001$) in the proportions of total nicotine metabolites eliminated as cotinine, which ranged from 6% to 31% (mean 15%).

### *Reported cigarette consumption and lung cancer risk*

Table I shows that overall self-reported current smokers had an odds ratio of 6.3 (95% CI 3.5-11.4) for risk of lung cancer as compared with those reported to be non-smokers. It also shows the much lower risk (odds ratio 1.3, 95% CI 0.4-4.2) of those smokers who reported smoking fewer than ten cigarettes a day.

### *TNM excretion and lung cancer risk*

The increasing risk of lung cancer as a function of increasing TNM/creatinine ratios among smokers is shown in Table II. Thus the odds ratios among the three tertiles were 0.9 (95% CI 0.2-3.1), 14.1 (95% CI 6.2-31.7) and 18.8 (95% CI 8.2-42.9) respectively.

### *Histological type and lung cancer risk in relation to smoking*

Data on histological type of lung cancer were available through the cancer registry for 49 of the patients (De Waard et al., 1995). Relative risk in relation to cotinine and TNM excretion was computed separately for adenocarcinoma in contrast to the sum of other histological types. The results are summarised in Table III and show that the relationship with smoking is much weaker for adenocarcinoma of the lung than for the other pulmonary cancers.

**Table I** Estimates of relative nicotine intake and risk of lung cancer according to reported cigarette consumption

| *Daily reported cigarette consumption* | *Number of subjects*[a] | *Positive urine test: n (%)* | *TNM*[b] *in positive samples: range (geometric mean)* | *Lung cancer cases/controls* | *Odds ratio (95% CI)* |
|---|---|---|---|---|---|
| Nil | 208 | 0 (0) | | 20/188 | 1.0 |
| <10 | 32 | 23 (72) | 1.6-22.4 (6.5) | 4/ 28 | 1.3 (0.4-4.2) |
| 10-20 | 57 | 56 (98) | 3.2-29.3 (13.2) | 29/ 28 | 9.7 (4.9-19.5) |
| >20 | 28 | 28 (100) | 4.4-25.6 (14.5) | 14/ 14 | 9.4 (3.9-22.5) |
| All smokers | 117 | 107 (91) | 1.6-29.3 (11.8) | 47/ 70 | 6.3 (3.5-11.4) |
| Total | 325 | 107 (33) | | 67/258 | |

[a]Subjects with missing reported cigarette consumptions as well as ex-smokers are excluded. [b]μg total nicotine metabolites $mg^{-1}$ creatinine. TNM, total nicotine metabolites.

**Table II** Risk of lung cancer according to urinary excretion of total nicotine metabolites in active smokers

| *Urinary TNM μg mg creatinine⁻¹ range (geometric mean)* | *Lung cancer cases* | *Controls* | *Odds ratio (95% CI)* |
|---|---|---|---|
| Non-smokers[a] | 21 | 197 | 1.0 |
| 1.6–9.9 (5.8) | 3 | 32 | 0.9 (0.2–3.1) |
| 10.1–17.6 (13.7) | 21 | 14 | 14.1 (6.2–31.7) |
| 17.7–29.3 (21.3) | 24 | 12 | 18.8 (8.2–42.9) |
| All smokers (12.0) | 48 | 58 | 7.8 (4.3–14.0) |
| Total | 69 | 255 | |

[a]Samples giving negative qualitative tests. TNM, total nicotine metabolites.

**Table III** Odds ratios in relation to TNM excretion for different types of lung cancer

| *TNM (μg mg⁻¹ creatinine)* | *OR adenocarcinomas (95% CI)* | *OR other carcinomas (95% CI)* |
|---|---|---|
| Non-smokers[a] | 1.0 | 1.0 |
| 1.6–9.9 | No OR estimate[b] | 1.0 (0.1–9.1) |
| 10.1–17.6 | 14.4 (2.5–81.3) | 22.0 (6.1–79.8) |
| 17.7–29.3 | 6.2 (1.4–26.6) | 17.6 (4.4–71.0) |
| All smokers | 4.9 (1.6–14.4) | 10.5 (3.8–29.3) |

[a]Samples giving negative qualitative tests. [b]No cases among (light) smokers.

### Discussion

The excellent results obtained using the simple qualitative diethylthiobarbituric acid extraction procedure to distinguish between active smokers on the one hand, and non-smokers and passive smokers on the other, confirms the original results obtained using the method (Peach *et al.*, 1985). It is noteworthy that the cut-off point for the qualitative test occurred at concomitant cotinine concentrations of about 100 μg $l^{-1}$, a level similar to the optimal cut-off point (about 70 μg $l^{-1}$) based on self-reported smoking status and urinary cotinine concentrations (De Waard *et al.*, 1995). These cut-off points are also similar to that (50 μg $l^{-1}$) recommended by Jarvis *et al.* (1987) and a value of about 128 μg $l^{-1}$ suggested by the results obtained by Wald *et al.* (1984). These findings therefore indicate that the simple, cheap diethylthiobarbituric acid extraction procedure, which has a potential throughput of greater than 60 samples per hour, is at least as efficient at identifying active smokers as the much more technically demanding cotinine-based procedures.

The results presented in Tables I and II show that the slightly higher odds ratio for the risk of lung cancer in biochemically proven smokers (7.8) as compared with a value of 6.3 for self-admitted smokers arose through the presence of a significant proportion (9%) of very light smokers. Since their nicotine intakes were similar to those of more heavily exposed passive smokers (De Waard *et al.*, 1995) it is suggested that they were probably non-addicted social smokers. By contrast with numerous other studies reported in the literature it is noteworthy that the women followed in our study were remarkably truthful in reporting their smoking status. Thus not a single active smoker was identified among the self-reported non-smokers. Such 'deceivers' (Jarvis *et al.*, 1987) can considerably complicate the interpretation of questionnaire-based epidemiological investigations and provide an important incentive for using the direct biochemical epidemiology approach. The absence of deceivers in our study may well be due to the fact that when the smoking histories were obtained (1975–83) there was much less public awareness of the health dangers of smoking.

Another reason that TNM/creatinine ratios should provide a much better estimate of smoke intake and therefore a steeper dose–response curve for lung cancer risk than self-reported cigarette consumption is the previously described evidence for the greatly differing efficiencies with which individuals smoke their cigarettes. To test whether this might have been the case, the biochemically proven active smokers were divided according to their TNM/creatinine ratios into unequal tertiles so as to match the proportions of smokers in the three self-reporting categories set out in Table I. However a chi-squared analysis showed that the dose–dependent risk relationship based on TNM/creatinine ratios [odds ratios of 1.1 (0.3–3.9), 8.6 (4.2–17.8) and 29.5 (11.3–77.2) respectively], was not significantly steeper ($P = 0.11$) than that based on cigarette consumptions [1.3 (0.4–4.2), 9.7 (4.9–19.5) and 9.4 (3.9–22.5) respectively].

A comparison of the odds ratios for lung cancer risk among equal tertiles of active smokers based on TNM/creatinine ratios set out in Table II (0.9, 14.1 and 18.8 respectively) with the corresponding odds ratios (1.3, 10.3 and 9.8 respectively) based on cotinine/creatinine ratios (De Waard *et al.*, 1995) shows that the simpler colorimetric method for estimating total nicotine metabolites performed at least as well in demonstrating the dose dependence of smoking-related lung cancer risk as the highly sophisticated GC–MS method for estimating cotinine.

The evidence obtained in our study demonstrating the large individual differences in the proportions of inhaled nicotine excreted as cotinine confirms the findings of Byrd *et al.* (1992) and Benowitz *et al.* (1994).

At first sight it might seem surprising that single estimates of urinary nicotine metabolite excretion were so predictive of subsequent lung cancer risk. However there is a considerable body of indirect evidence to suggest that once the smoking habit is firmly established daily individual nicotine intakes are likely to continue for many years with little change. Thus numerous studies have shown that when individuals change to smoking brands of cigarettes with reduced nicotine yields they rapidly alter their mode of smoking (compensate) so as to obtain their former accustomed daily nicotine intakes (for references see Withey *et al.*, 1992). The fact that urinary TNM/creatinine ratios of samples obtained when the cohort was enrolled into the study correlated well with those obtained a year later provides objective evidence for the assumption that these reflected the chronic smoking habits of the women in the cohort. Furthermore, the fact that the tar–nicotine ratios of most brands of cigarettes are very similar (Phillips and Waller, 1991), implies that intakes of carcinogenic tar will be closely related to those of nicotine.

The overall odds ratio of 7.8 for biochemically proven smokers (Table II) confirms the cotinine-based evidence presented in our companion paper (De Waard *et al.*, 1995) and supports the conclusion of Garfinkel and Stellman (1988) that after allowing for the duration of smoking, women probably have a lung cancer risk of similar magnitude to that encountered by men.

The results presented in Table III confirm the cotinine-based findings reported and discussed in our companion paper (De Waard *et al*., 1995) that the relationship between smoking and adenocarcinoma of the lung is much weaker than that of other histological types.

The biochemical epidemiology approach used in this investigation to explore the relationship between smoking and lung cancer could profitably be employed in the study of other smoking-related conditions such as ischaemic heart disease, and also to overall mortality in situations where appropriate urine banks are available. The great advantage of this approach is that it does not require either histories of active smoking or evidence concerning potential exposure to environmental tobacco smoke.

Urine samples can be rapidly tested by the qualitative diethylthiobarbituric acid extraction procedure (Peach *et al*., 1985) and the dose dependence of disease in the active smokers explored by determining the TNM/creatinine ratios by the automated colorimetric method of Puhakainen *et al*. (1987). The possible influence of passive smoking can then be assessed using cotinine-based assays (Wald *et al*., 1984; De Waard *et al*., 1995).

**Acknowledgements**
The authors are indebted to J Fracheboud, to FJJ Bosman and CHF Gimbrère of the Comprehensive Cancer Centre (IKMN) at Utrecht for providing data on lung cancer mortality and incidence respectively. The project was subsidised partly by the Prevention Fund (No. 28-2154), the Hague and by the Dutch Chief Medical Inspectorate of Health.

### References

ARMITAGE AK, ALEXANDER J, HOPKINS R AND WARD C. (1988). Evaluation of a low to middle tar/medium nicotine cigarette designed to maintain nicotine delivery to the smoker. *Psychopharmacology*, **96**, 447–453.

BARLOW RD, STONE RB, WALD NJ AND PUHAKAINEN EVJ. (1987). The direct barbituric acid assay for nicotine metabolites in urine: a simple colorimetric test for routine assessment of smoking status and cigarette intake. *Clin. Chim. Acta*, **165**, 45–52.

BENOWITZ NL, HALL SM, HERNING RI, JACOB P, JONES RT AND OSMAN AL. (1983). Smokers of low-yield cigarettes do not consume less nicotine. *New Engl. J. Med*., **309**, 139–142.

BENOWITZ NL AND JACOB P. (1984). Daily intake of nicotine during cigarette smoking. *Clin. Pharmacol. Ther*., **35**, 499–504.

BENOWITZ NL, JACOB P, FONG I AND GUPTA S. (1994). Nicotine metabolic profile in man: comparison of cigarette smoking and transdermal nicotine. *J. Pharmacol. Exp. Ther*., **268**, 296–303.

BYRD G, CHANG KM, GREENE JM AND DE BETHIZY JD. (1992). Evidence for urinary excretion of glucuronide conjugates of nicotine, cotinine and *trans-3'*-hydroxycotinine in smokers. *Drug Metab. Dispos*., **20**, 192–197.

DE WAARD F, KEMMEREN JM, VAN GINKEL LA AND STOLKER AAM. (1995). Urinary cotinine and lung cancer risk in a female cohort. *Br. J. Cancer*, **72**, 784–787.

GARFINKEL L AND STELLMAN SD. (1988). Smoking and lung cancer in women: findings in a prospective study. *Cancer Res*., **48**, 6951–6955.

HILL P, HALEY NJ AND WYNDER EL. (1983). Cigarette smoking: carboxyhemoglobin, plasma nicotine, cotinine and thiocyanate *vs* self-reported smoking data and cardiovascular disease. *J. Chron. Dis*., **36**, 439–449.

JARVIS MJ, TUNSTALL-PEDOE H, FEYERABEND C, VESEY C AND SALLOOJEE Y. (1984). Biochemical markers of smoke absorption and self-reported exposure to passive smoking. *J. Epidemiol. Community Health*, **38**, 335–339.

JARVIS MJ, TUNSTALL-PEDOE H, FEYERABEND C, VESEY C AND SALOOJEE Y. (1987). Comparison of tests used to distinguish smokers from nonsmokers. *Am. J. Public Health*, **77**, 1435–1438.

PEACH H, ELLARD GA, JENNER PJ AND MORRIS RW. (1985). A simple, inexpensive urine test of smoking. *Thorax*, **40**, 351–357.

PHILLIPS GF AND WALLER RE. (1991). Yields of tar and other smoke components from UK cigarettes. *Fd. Chem. Toxicol*., **29**, 469–474.

PUHAKAINEN EVJ, BARLOW RD AND SALONEN JT. (1987). An automated colorimetric assay for urine nicotine metabolites: a suitable alternative to cotinine assays for the assessment of smoking status. *Clin. Chim. Acta*, **170**, 255–262.

RUSSELL MAH, JARVIS MJ, FEYERABEND C AND SALOOJEE Y. (1986). Reduction of tar, nicotine and carbon monoxide intake in low tar smokers. *J. Epidemiol. Community Health*, **40**, 80–85.

WALD NJ, BOREHAM J, BAILEY A, RITCHIE C, HADDOW JE AND KNIGHT G. (1984). Urinary cotinine as marker of breathing other people's tobacco smoke. *Lancet*, **1**, 230–231.

WALL MA, JOHNSON J, JACOB P AND BENOWITZ NL. (1988). Cotinine in the serum, saliva and urine of nonsmokers, passive smokers and active smokers. *Am. J. Public Health*, **78**, 699–701.

WITHEY CH, PAPACOSTA AO, SWAN AV, FITZSIMMONS BA, ELLARD GA, BURNEY PGJ, COLLEY JRT AND HOLLAND WW. (1992). Respiratory effects of lowering tar and nicotine levels of cigarettes smoked by young male middle tar smokers. II. Results of a randomised controlled trial. *J. Epidemiol. Community Health*, **46**, 281–285.

WOODWARD M, TUNSTALL-PEDOE H, SMITH WCS AND TAVENDALE R. (1991). Smoking characteristics and inhalation biochemistry in the Scottish population. *J. Clin. Epidemiol*., **44**, 1405–1410.